# Family-Centered Rehabilitation Care for Children with Cerebral Palsy in Saudi Arabia: Perceived Helpfulness of Formal and Informal Family Support

**DOI:** 10.3390/healthcare14101282

**Published:** 2026-05-08

**Authors:** Ahmad A. Alharbi, Abdulaziz A. Albalwi, Hamad S. Al Amer, Samia A. Alamrani, Hani F. Albalawi, Nihad A. Almasri, Maysoun N. Saleh

**Affiliations:** 1 Department of Health Rehabilitation Sciences, Faculty of Applied Medical Sciences, University of Tabuk, Tabuk 71491, Saudi Arabia; aa-albalwi@ut.edu.sa (A.A.A.); halamer@ut.edu.sa (H.S.A.A.); salamrani@ut.edu.sa (S.A.A.); hf_albalawi@ut.edu.sa (H.F.A.); m-saleh@ut.edu.sa (M.N.S.); 2 Department of Rehabilitation Sciences, College of Health Sciences, QU Health Qatar University, Doha P.O. Box 2713, Qatar; nalmasri@qu.edu.qa; 3 Department of Physiotherapy, School of Rehabilitation Sciences, The University of Jordan, Amman 11942, Jordan

**Keywords:** cerebral palsy, family-centered care, rehabilitation, informal support, formal support

## Abstract

**Background/Objectives**: Within a family-centered care framework, families of children with cerebral palsy (CP) rely on both informal and formal support systems to manage caregiving demands, yet the adequacy and perceived helpfulness of this support vary. This study aimed to examine the availability of different support sources for families of children with CP in Saudi Arabia, explore their perceived helpfulness, and evaluate the influence of child-, parent-, geographical-, and household-related characteristics on the level of family support helpfulness. **Methods**: A cross-sectional study was conducted among 223 caregivers of children diagnosed with CP across Saudi Arabia. The participants completed the Child and Family Questionnaire and the Family Support Scale (FSS), and the children were classified on the Gross Motor Function Classification System (GMFCS). Kruskal–Wallis *H* and Mann–Whitney *U* tests examined the differences in perceived support helpfulness across family, child, and household characteristics. **Results**: Spousal and kinship support were perceived as the most helpful sources, with families of children with bilateral spastic CP and more severe GMFCS perceiving less support. Overall, informal social support was significantly perceived as more helpful than formal support. **Conclusions**: Families of children with CP rely mainly on spouses and relatives for support, with lower perceived helpfulness among those facing greater caregiving demands, highlighting the need to improve the accessibility, coordination, and quality of support services.

## 1. Introduction

Cerebral palsy (CP) is one of the most common childhood disabilities worldwide. In Arabic-speaking countries. The prevalence of CP is 1.8 per 1000 live births, whereas the prevalence is even higher in Saudi Arabia (2.34 per 1000 live births) [1,2]. CP is defined as a permanent brain disorder that causes a range of motor impairments, potentially resulting in atypical postures, activity limitations, and participation restrictions. While primarily a motor disorder, CP can also affect sensation, cognition, communication, perception, and emotional and behavioral functioning [3].

Families of children with disabilities, particularly those with CP, play a central role in the rehabilitation process [4,5]. Their active involvement in daily care, implementation of therapeutic exercises, and coordination of healthcare and educational services is essential for maintaining the continuity of care beyond clinical settings and supporting the child’s functional development [4,5]. This collaborative involvement is conceptualized within family-centered care, an approach that emphasizes partnership between families and healthcare professionals in planning and delivering care. Evidence shows that engaging parents as partners enhances treatment adherence, functional outcomes, and caregiver satisfaction [6,7]. However, this level of involvement requires appropriate and sustained support to enable families to maintain their caregiving role effectively.

Previous studies have shown that families of children with CP frequently report a need for both formal and informal support resources [4,5]. Formal support includes services provided by medical, rehabilitation, educational, and social institutions, whereas informal support stems from family, kinship networks, friends, and broader community ties [8]. Bertule and Vetra (2020) found that family needs in Latvia were primarily influenced by socio-economic conditions and the availability of support from peers, family members, and professionals [5]. Almasri et al. (2014) reported that professional helpers, such as therapists, offered more significant support to children with CP compared to other informal support sources and professional agencies [9]. Informal support for families of children with CP is associated with better family functioning and caregiver psychological well-being [10]. Family members—including parents, siblings, and grandparents—play key caregiving and developmental roles [11,12,13,14,15], while support from friends and neighbors enhances emotional well-being and parental management; formal support further contributes to family resilience [16,17].

The amount and quality of support that families receive are influenced by multiple interacting factors. Dunst et al. (1984) reported that child, parent, and family outcomes are empirically associated with several variables, including child attributes, environmental features, parenting behaviors and practices, family and social system characteristics, and practitioner-related measures [8]. Almasri et al. (2014) recommended that service providers consider parents’ age, children’s age, and motor abilities when advising families on support options tailored to their needs [9]. Geographical area is another factor shaping family support, as parents of children with disabilities often struggle to find childcare services for their school-age children within their local region [18]. Geographical variation also contributes significantly to unmet needs, as the availability and accessibility of services differ across regions [19]. Moreover, household-related variables, such as family size and income, play a crucial role in determining the effectiveness of support systems [20].

Child-related factors, particularly the severity of motor disability and mobility limitations, strongly influence families’ support needs. Studies have consistently shown that parents of children who require wheeled mobility (Gross Motor Function Classification System (GMFCS) levels IV and V) report greater needs for financial, community, and family support than those of children who are ambulatory (GMFCS I, II, and III) [4,20]. Parents of children with severe motor limitations tend to express higher demands for information, community services, and emotional support, underscoring the need for tailored support services [5].

Research on families of children with CP in Saudi Arabia shows uneven availability across support sources [21,22]. Kinship support is culturally prominent but often insufficient to meet families’ physical and financial needs, while spousal support is reported but remains underexplored in the literature [21,22]. Informal support from friends and wider community is limited, partly due to social stigma and reliance on close family networks, which may contribute to isolation [22,23]. Community programs and organizational support exist but appear limited in scope and accessibility [22]. Although professional services are available, families report challenges in access, navigation, and continuity of care, particularly outside major urban areas, along with gaps in psychological and family-centered services [21,24,25].

In Saudi Arabia, to date, no study has explored sources of support available to families of children with CP despite the high prevalence of CP. This study aimed to 1—examine the availability of different sources of support to families of children with CP in Saudi Arabia, 2—explore the helpfulness of available sources of support, and 3—to evaluate the influence of child-, parent-, geographical-, and household-related characteristics on the level of helpfulness of family support. Accordingly, this study sought to answer these research questions: What sources of support are available to families of children with CP in Saudi Arabia? And to what extent do child-related, parent-related, and geographical/household characteristics influence families’ perceptions of the helpfulness of the received support during the rehabilitation journey of children with CP in Saudi Arabia? Based on the existing evidence, it was hypothesized that child-related, parent-related, and geographical/household characteristics would significantly affect families’ perceptions of the helpfulness of support provided.

## 2. Materials and Methods

### 2.1. Study Design and Ethical Considerations

This study was part of cross-sectional research conducted across multiple regions in Saudi Arabia that explored the characteristics of children with CP and the services they received [26]. The study protocol adhered to the ethical rules and regulations of the National Committee of Bioethics (NCBE) and received approval from the local Ethics Committee of the University of Tabuk (UT-175-39-2022). Written informed consent was obtained from parents upon their agreement to participate. Although the recruitment was conducted within clinical care settings, several procedures were considered to minimize any perceived coercion and ensure voluntary participation. Research assistants (RAs) were not the treating the therapists of the targeted participants. This separation between clinical care and research roles was maintained to reduce any potential influence. Families were informed that participation was entirely voluntary, that their decision would not affect the care they receive, and that they can withdraw at any time during the study. The physical therapists who provide patients’ care were not involved in obtaining consent or collecting data; in addition, this study was reported in accordance with the STROBE Statement (Strengthening the Reporting of Observational Studies in Epidemiology). All relevant items from the STROBE checklist were addressed to ensure transparency and completeness in the reporting of study design, participant selection, variables, data sources, and statistical analyses.

### 2.2. Participants

The required sample size was estimated using a standard formula for proportions in cross-sectional studies, assuming a 95% confidence level (*Z* = 1.96), a 5% margin of error (*d* = 0.05), and a conservative population proportion of 50% (*p* = 0.50). The assumption of a 50% response distribution was used as it yields the maximum required sample size, providing a conservative estimate in the absence of prior data. Accordingly, a minimum sample of 385 participants was required. However, this study included a sample of convenience of 223 children diagnosed with CP and their parents. This achieved sample size yielded an acceptable margin of error of approximately ±6.5% at the 95% confidence level, supporting the adequacy of the sample for exploratory and descriptive analysis.

Participants were recruited from eight of the 13 provinces in Saudi Arabia, (total of 14 cities). For analytical purposes, the provinces were grouped into five geographic regions—North, South, Central, East, and West—consistent with common regional classifications in Saudi Arabia. The Central region includes the capital city, Riyadh; the Western region includes the holy cities and is a major center for religious tourism; the Eastern region is characterized by the oil industry; and the Northern and Southern regions are primarily agricultural.

Children were eligible for inclusion if they met the following criteria: (1) they were aged ≤ 18 years; (2) they had a medical diagnosis of CP confirmed by a neuro-pediatrician; (3) they were receiving services from one of the selected centers or hospitals within the five districts in Saudi Arabia; and (4) they had parental consent. Children were excluded if they had another genetic, neurologic, or pervasive developmental disorder (such as Down syndrome, spina bifida, or autism) other than CP.

### 2.3. Measures

#### 2.3.1. Child and Family Questionnaire

An electronic survey was developed by the research team to gather demographic data on participating children and their families, including child and parent age, secondary health problems, past medical and surgical history, school system attended, parental educational level, employment status, family income, and geographic region of residence.

#### 2.3.2. Gross Motor Function Classification System—Expanded and Revised

The Gross Motor Function Classification System—Expanded and Revised (GMFCS-E&R) is a widely used, valid, and reliable tool for classifying gross motor function in children with CP [27,28]. This five-level system categorizes functional abilities related to sitting, transfers, and mobility, considering functional limitations and the use of assistive devices. Level I describes children who walk without limitations, whereas Level V refers to those who require transportation in a manual wheelchair. The GMFCS has demonstrated strong content, construct, and discriminative validity, along with high inter-rater reliability [28,29]. The GMFCS levels for participating children were determined by pediatric physical therapists who were trained by the researchers on its use.

#### 2.3.3. Family Support Scale (FSS)

The Family Support Scale (FSS) assesses the perceived helpfulness of various sources of support for families with young children [9]. The FSS consists of 19 items rated on a 5-point scale ranging from not at all helpful (1) to extremely helpful (5), with an additional N/A option if a source of support is unavailable to the family. Items are grouped into five subscales: professional services (4 items), program organizations (4 items), informal support (6 items), spouse/partner support (3 items), and kinship (2 items). The combined score of four informal subscales—program organizations, informal support, spouse/partner support, and kinship—represents the informal support score, whereas the professional services subscale reflects the formal support score. The total FSS score is obtained by summing all 19 item ratings. The Arabic version of the FSS (A-FSS) has been shown to be valid and reliable for Arabic-speaking families of children with CP [9].

### 2.4. Procedure

Twelve trained RAs, all of whom were physical therapists, attended a 2-h session to review the study procedures and measurement tools before data collection commenced. The RAs approached parents during their child’s routine physical therapy appointments at the participating hospitals or centers. After explaining the study’s aims, procedures, and expected outcomes, written informed consent was obtained from parents who agreed to participate. Each participant completed a structured interview conducted by an RA, which included the Child and Family Questionnaire and the FSS, followed by a GMFCS assessment of the child. The entire interview and assessment process took approximately 1 h.

### 2.5. Data Analysis

For data analysis, children were categorized into two groups based on GMFCS levels: ambulatory (levels I–III, with or without limitations) and non-ambulatory (levels IV–V). In addition, children with spastic hemiplegia and monoplegia were grouped under unilateral spastic CP, whereas bilateral spastic CP included children with diplegia, triplegia and quadriplegia. The provincial residences were grouped into five geographic regions: North, South, Central, East, and West. Maternal and paternal education levels were grouped as low (less than high school), intermediate (high school or college), or high (Bachelor’s degree or higher).

Descriptive statistics—including means, standard deviations, ranges, frequencies, and percentages—were used to summarize participant characteristics and their FSS scores. The proportion of participants indicating that a given source of support was unavailable (i.e., rated as “NA”) was calculated to reflect support availability. For each support subscale, participants who rated all constituent items as “NA” were classified as not having access to that type of support. Statistical analyses assessed differences across sources of support (kinship, spouse, informal, programs/organizations, and professional services) and between informal social support and formal support. Additionally, we examined the influence of child-, parent-, geographical-, and household-related characteristics on informal social support, formal support, and total support levels. Outcome variables included the total FSS score, informal social and formal support scores.

The distribution of outcome variables was assessed for normality using the Shapiro–Wilk and Kolmogorov–Smirnov tests. The results from both tests indicated that all variables deviated from a normal distribution (*p* > 0.05). Accordingly, non-parametric statistical methods were applied, including the Friedman test, Wilcoxon signed-rank test, Kruskal–Wallis *H* test, and Mann–Whitney *U* test, to examine differences between variables.

To examine differences among the FSS subscale scores, the Friedman test was performed, followed by pairwise comparisons using the Wilcoxon signed-rank test. The differences between informal social support and formal support were also evaluated using the Wilcoxon signed-rank test.

To examine the influence of participant characteristics on informal social support, formal support, and total FSS scores, Mann–Whitney *U* tests were used for binary variables, and Kruskal–Wallis *H* tests were used for variables with three or more levels. When the Kruskal–Wallis *H* test results were significant, post hoc comparisons were conducted using the Mann–Whitney *U* test.

Effect sizes (ES) were estimated using effect size correlation coefficient (*r*) for the Wilcoxon signed-rank and Mann–Whitney *U* tests, epsilon squared (*ε*^2^) for the Kruskal–Wallis *H* test, and Kendall’s W for the Friedman test. ES values were interpreted using the following thresholds: *r* and Kendall’s W values of ≥0.10, ≥0.30, and ≥0.50 were considered small, medium, and large, respectively; and *ε*^2^ values of ≥0.01, ≥0.06, and ≥0.14 indicated small, medium, and large ES [30].

All statistical analyses were conducted using IBM SPSS Statistics software (Version 25.0; IBM Corp., Armonk, NY, USA) with the significance level set at α = 0.05. To control for type I error in multiple comparisons, the Bonferroni correction was applied by adjusting the significance level according to the number of comparisons.

## 3. Results

### 3.1. Participants’ Characteristics

The demographic characteristics of families and children in this study are outlined in Table 1. The respondents were 81.6% mothers and 18.4% fathers or other family members. Children aged 12 years or older were less represented in the sample (8.5%), and boys (58.7%) outnumbered girls. Spastic CP was the most common type (79.8%), bilateral spastic CP (67.7%) being the most frequent distribution. More than half of the children (55.2%) were in the ambulatory group of GMFCS.

Regarding parents, most mothers were 40 years old or younger, had a high school education or higher, and were unemployed (82.1%). The majority of fathers were between 31 and 50 years old, had a high school education or higher, and were employed full-time (76.2%). In terms of geographical and household characteristics, most participant children resided in the Western, Southern or Central regions, and lived with both parents (94.6%). The majority of families were in the middle-income bracket. According to the General Authority for Statistics, the average monthly income in Saudi Arabia is 10,238 SAR [31].

### 3.2. Differences in the Availability and Helpfulness of Various Sources of Support

Table 2 shows the availability of the five major sources of support for families, and the N/A score on the scale indicated that the source was not available. The most available sources of support for families were spouse and kinship, while program organizations and professional services were the least available services.

A more detailed examination of individual support sources revealed that, within program-based organizations, the most frequently reported as unavailable were parent group members (145; 65%), social groups or clubs (164; 73.5%), and school or daycare centers (160; 71.7%). Within professional services, early childhood intervention programs were most commonly reported as unavailable (184; 82.5%), followed by professional agencies (143; 64%). Additionally, among informal support sources, support from religious associations was most frequently reported as unavailable (180; 80.7%).

Regarding the mean scores of the FSS and its subscales (Table 3), the average total FSS score was 24.3 ± 17.7 (range 0–78). Informal social support was significantly higher than formal support, with mean scores of 6.3 ± 4.2 and 3.8 ± 4.3, respectively (*p* < 0.005, *r* = 0.64). Among the subscales, kinship (2.3 ± 1.8) and spouse support (2.3 ± 1.5) had the highest mean scores, whereas programs/organizations (0.5 ± 0.8) and professional services (0.9 ± 1.1) had the lowest. The Friedman test revealed a statistically significant difference among the five sources of support (*p* < 0.005, W = 0.48). Pairwise comparisons were significant for all sources (*p* < 0.005) except between kinship and spouse support (*p* = 0.084, *r* = 0.13) (Supplementary Table S1).

### 3.3. Influence of Participants’ Characteristics on the Level of Helpfulness of Support

#### 3.3.1. Child-Related Characteristics

Table 4 shows the influence of child-related factors on support helpfulness levels. Child age and sex did not affect the level of helpfulness of support as rated by parents. However, significant differences were observed with respect to CP distribution, with families of children with unilateral spastic CP reporting higher helpfulness of support from informal social sources (*p* = 0.009, *r* = 0.20), formal sources (*p* = 0.012, *r* = 0.19), and total support (*p* = 0.005, *r* = 0.21). Furthermore, the ambulatory group (GMFCS I-III) was significantly associated with higher levels of helpfulness of support from formal sources (*p* = 0.018, *r* = 0.16) and informal social sources (*p* = 0.010, *r* =0.17), as well as a higher overall support score (*p* = 0.007, *r* = 0.18).

#### 3.3.2. Parent-Related Characteristics

The influence of parent-related factors on levels of support is presented in Table 5. Both mother’s age (*p* = 0.023, *ε*^2^ = 0.03) and father’s age (*p* = 0.035, *ε*^2^ = 0.03) had a significant effect on levels of informal social support. However, following Bonferroni-adjusted post hoc comparisons at α = 0.0086, no statistically significant pairwise differences were observed for mother’s age, with *p*-values ranging from 0.009, *r* = 0.24 (21–30 years vs. 41–50 years) to 0.667, *r* = 0.06 (41–50 years vs. >51 years). In contrast, for father’s age, a single statistically significant difference was identified between the 31–40 years and 41–50 years groups (*p* = 0.003, *r* = 0.22), while all other comparisons were not significant.

#### 3.3.3. Geographical and Household Characteristics

Table 6 presents the results of the Kruskal–Wallis *H* test and Mann–Whitney *U* test examining the influence of geographical and household factors on support levels. Geographic region was a significant factor for both formal and informal social support, as well as for the overall support score (*p* < 0.005, *ε*^2^ = 0.10–0.13). Specifically, post hoc analyses (Supplementary Table S2) revealed that families residing in the Central region reported significantly more helpfulness of informal, formal, and total support compared with families in the Eastern and Northern regions (*p* < 0.005, *r* = 0.46–0.63). The Western region also showed higher helpfulness of support than the Northern region in formal (*p* = 0.003) and total support (*p* = 0.004), as well as higher formal support than the Eastern region (*p* = 0.002, *r* = 0.29). Additional differences included higher helpfulness of informal and total support in the Central region compared with the Western region (*p* < 0.005, *r* = 0.40 and 0.38, respectively), and higher total support in the Eastern region compared with the Northern region (*p* = 0.005, *r* = 0.47). No significant differences were found between the Southern and Western regions or between the Southern and Central regions across all sources of support, between the Eastern and Northern regions for informal and formal support, or between the Central and Western regions for formal support.

Finally, Mann–Whitney *U* test results showed that single mothers reported significantly higher helpfulness of support from formal sources compared to two-parent families (*p* = 0.019, *r* = 0.16). Additionally, the presence of other children with special needs in the household was significantly associated with lower perception of helpfulness of support from formal sources (*p* = 0.019, *r* = 0.16) and total support (*p* = 0.028, *r* = 0.15).

## 4. Discussion

Within a family-centered care framework, the substantial caregiving demands faced by families of children with disabilities necessitated access to comprehensive, coordinated support systems that recognize families as central partners in care [4,5]. Both formal and informal sources of support play a critical role in promoting family well-being and quality of life, and ultimately shaping child outcomes, underscoring the importance of responsive, family-driven rehabilitation services [32,33,34].

This was the first study to examine available sources of support and their perceived helpfulness for families of children with CP in Saudi Arabia. The most helpful sources of support were the spouse and kinship networks. These findings align with those of Almasri et al. (2014) who explored family support in a similar Arabic cultural context and emphasized the importance of kinship and spouse support for families of children with CP [9]. Our data showed no significant differences between these two sources, further reinforcing that both are closely related and highly helpful for these families. Pfeifer et al. (2014) also reported that core and extended family members (e.g., spouse, mother, and siblings) and friends are the most common providers of support for caregivers of children with CP in Brazil [20]. However, in a culturally different context, McIntyre and Brown (2018) found that American families of children with autism used both formal and informal support and perceived both to be helpful [35]. These differences may reflect cultural variation, differences in health care systems, or differences in the type of child disability.

Other informal sources of support were either not available or, when available, were perceived as not helpful by most participants. In Saudi culture—and in Arab cultural contexts more broadly [9]—both nuclear and extended family assume a central social role. Informal sources, including neighbors, religious groups, and schools or daycare centers, may be unaware of the child’s diagnosis (probably because of family’s fear of stigma), which limits their ability to support families caring for a child with CP. As such, lower perceived helpfulness may reflect reduced access, awareness, or utilization, rather than a true lack of benefit.

Professional or formal support was among the most frequently reported categories as either not available or not helpful. This finding may reflect restricted access to services and limited engagement with providers, both of which can shape families’ perceptions of usefulness. However, this interpretation should be made with caution. The instrument used does not distinguish whether a “Not Available” response indicates a true absence of services or the presence of services that families were unable to access. This represents an inherent limitation of the measure and restricts the ability to draw definitive conclusions about availability versus accessibility, which could contribute to discrepancies between perceived and delivered support.

In addition, it is important to consider that some forms of support may be implicit or “hidden” within routine care processes. Support delivered through ongoing clinical interactions, guidance during therapy sessions, or informal communication with healthcare providers may not be explicitly recognized by families as distinct sources of support and, therefore, may be underreported in structured instruments. From a methodological perspective, this aligns with prior work highlighting the challenges of interpreting structured health data when underlying constructs are not fully differentiated [36]. Specifically, response categories may capture heterogeneous underlying experiences; thus, ratings such as “Not Available” or low perceived helpfulness may reflect not only absence of services, but also limited access, variable engagement, or support embedded within routine care that is not perceived as a separate resource.

Nonetheless, the reported absence or limited perceived helpfulness of professional/formal support remains of particular concern given the critical role of formal rehabilitation services during the early stages following diagnosis, when families require expert guidance regarding the child’s medical, educational, and developmental needs [20]. Physical therapists, often serving as the first point of professional contact, play a central role in delivering family-centered care through assessment, collaborative goal setting, and home-based intervention. Evidence indicates that family-centered approaches enhance family satisfaction, reduce stress, and improve adherence to therapy [6]. Conversely, when care is perceived as directive or lacking personalization, families may rate professional support as less helpful, even during these crucial early stages [37].

Overall, families of children with disabilities face substantial caregiving demands, making access to both formal and informal support essential for family well-being, parental mental health, and quality of life, as well as child outcomes [4,5,8,10,16,17,38].

Interestingly, families of children with less severe forms of CP (unilateral spastic CP) reported significantly higher levels of perceived helpfulness of support than families of children with more severe involvement (bilateral spastic CP). Similarly, families of ambulatory children (GMFCS levels I–III) reported greater perceived support compared to those with more limited functional mobility (GMFCS levels IV–V). While this pattern may appear counterintuitive, these findings likely reflect differences in families’ experiences and perceptions of support rather than objective differences in service provision. Families of children with higher levels of impairment may encounter challenges that influence how available support is perceived, including increased caregiving demands, social isolation, and stigma [39,40]. These factors have been associated with higher stress and lower perceived social support among caregivers of children with CP [10], which may contribute to lower helpfulness ratings.

Accordingly, the findings highlight the importance of family-centered rehabilitation approaches that prioritize meaningful engagement with families, collaborative goal setting, and responsiveness to varying functional needs [6]. Families of children with more severe CP may benefit from tailored support strategies, including enhanced guidance, caregiver training, and ongoing professional support to better align services with their perceived needs [41].

Notably, while motor function disability (GMFCS level and CP distribution) significantly affected family’s perception of support helpfulness, age did not appear to impact support requirements—a finding also reported in previous studies [4,20,42]. These findings highlight the importance of recognizing the specific challenges faced by families based on the child’s motor function rather than age, ensuring that professional support systems, including rehabilitation services, are appropriately targeted and effectively address their unique needs. However, it is important to note that our sample primarily included children under 12 years. Further research is warranted to examine family support and its influencing factors among families of adolescents with CP.

Regarding parent-related characteristics, there were no highly significant effects of any characteristic on the perception of helpfulness of support. Previous research in Saudi Arabia indicated that mothers of children with CP perceived available services as insufficiently family-centered and reported an unmet need for more comprehensive information about their children [24]. Mother’s age was not a determinant of perceived family-centeredness of services [24].

Geographic region was significantly associated with variations in perceived support, both in overall levels and across specific sources. These differences likely reflect variation in families’ experiences and perceptions of support across regions rather than definitive differences in service provision. Previous studies have reported regional variation in the utilization of rehabilitation services and in parent-reported unmet needs among families of children with disabilities [19,26], which may influence how support is perceived and evaluated. However, findings in our study should be interpreted cautiously, as some subgroup comparisons were based on small sample sizes, which may affect the reliability of the observed differences.

In addition, contextual factors such as household income and maternal education may shape families’ ability to engage with and benefit from available services, thereby influencing perceived helpfulness [43]. Families facing greater caregiving demands or logistical challenges may perceive support as less helpful, even when services are present. Given the perception-based and cross-sectional nature of this study, these findings should not be interpreted as direct evidence of regional disparities in service availability. Further research is needed to better understand the factors underlying geographic variation in perceived social support within the Saudi context.

Other household factors significantly influenced the perceived helpfulness of support in our study, such as being a single mother. This circumstance may have prompted greater assistance from both family networks and professionals, given the considerable demands of caring for a child with a disability as a single parent. In contrast, families with additional children with disabilities reported receiving less support, which may partly reflect their higher needs and expectations that lead them to perceive the available support as insufficient or less helpful.

Although the estimated sample size was 385, the study included 223 participants. This remains acceptable given the conservative assumptions used in the calculation, the inclusion of participants from multiple regions, and consistency with similar studies [5,20,22]. However, the smaller sample size may reduce estimate precision, limit generalizability, and decrease the ability to detect small subgroup differences.

In addition, some geographic areas (particularly northern and eastern) were underrepresented, which may have influenced findings related to geographic variation. In addition, some subgroup analyses were based on small sample sizes, limiting the stability and generalizability of these comparisons; thus, the reported effect sizes should be interpreted with caution. Furthermore, the relatively small number of adolescents limits the applicability of the findings to this group. Future research should include larger, more regionally balanced samples and greater representation of adolescents with CP to enhance generalizability.

The study used a univariate analytical approach due to its exploratory design and the distributional characteristics of the data, including non-normality and small subgroup sizes. While this allowed for the examination of group differences, it did not account for potential confounding factors. Future research using larger samples and multivariable analysis is recommended to identify independent predictors of support and provide a more comprehensive understanding of the factors influencing support.

Finally, the cross-sectional design of this study limits the ability to infer causal relationships; thus, findings should be interpreted as reflecting families’ perceptions of support helpfulness rather than system-level factors such as service availability or access. Future longitudinal studies are warranted to better examine temporal associations between variables. Additionally, families were not involved as research partners in the design or conduct of this study, and their inclusion in future research would better capture lived experiences. It is also important to note that a substantial proportion of support—particularly relational, educational, and care coordination activities—may be embedded within routine clinical interactions and therefore not fully captured by self-report measures. Future studies incorporating qualitative approaches or analyses of clinical documentation may help identify and better characterize this “hidden” layer of care, providing a more comprehensive understanding of family support.

## 5. Conclusions

Families of children with CP reported variability in the perceived helpfulness of different sources of support, with spouses and extended family remaining the most relied upon. Lower perceived helpfulness of both informal and formal support was more evident among families of children with more severe motor impairments and those caring for multiple children with disabilities, highlighting the importance of considering diverse family experiences and needs. These findings underscore the need for approaches that enhance meaningful engagement with families and tailor support to their perceived priorities. Future research should further explore factors influencing perceived support and develop strategies to better capture and address the full spectrum of support experienced by families of children with CP.

## Figures and Tables

**Table 1:** Demographic and background characteristics of the participants (*n* = 223).

Child-Related			Parent-Related			Geographical and Household		
Characteristics	*n*	%	Characteristics	*n*	%	Characteristics	*n*	%
**Age (years)**			**Mother’s Age** (years)			**Region**		
<3	47	21.1	21–30	72	32.3	Southern	51	22.9
3 to <6	73	32.7	31–40	99	44.4	Central	49	22.0
6 to <12	84	37.7	41–50	46	20.6	Eastern	28	12.6
≥12	19	8.5	>51	6	2.7	Western	88	39.5
						Northern	7	3.1
**Gender**			Mother’s Education *			**Living with**		
Boys	131	58.7	Low	42	18.8	Mother	11	4.9
Girls	92	41.3	Intermediate	97	43.5	Father	0	0.0
			High	84	37.7	Both parents	211	94.6
						Others	1	0.4
**Type of CP**			**Mother’s Employment**			**Children in the Same House**		
Ataxic	5	2.2	Unemployed	183	82.1	≤2	108	48.4
Dyskinetic	6	2.7	Part-time	5	2.2	3–4	89	39.9
Hypotonia	19	8.5	Full-time	35	15.7	≥5	26	11.7
Mixed **	7	3.1						
Spastic	178	79.8						
Unknown	8	3.6						
**Distribution of spastic CP** (***n*** = 178)			**Father’s Age** (years)			**Other Children with Special Needs in the Same House**		
Unilateral	27	12.1	21–30	19	8.5	No	190	85.2
Bilateral	151	67.7	31–40	108	48.4	Yes	33	14.8
			41–50	69	30.9			
			>51	27	12.1			
**GMFCS level**			Father’s Education *			**Monthly Income** (SR)		
I–III (Ambulatory)	123	55.2	Low	36	16.1	<5000	42	18.8
IV–V (Non-ambulatory)	100	44.8	Intermediate	111	49.8	5000–10,000	97	43.5
			High	76	34.1	10,001–15,000	48	21.5
						>15,000	36	16.1
			**Father’s Employment**					
			Unemployed	44	19.7			
			Part-time	9	4.0			
			Full-time	170	76.2			

CP, cerebral palsy; GMFCS, Gross Motor Function Classification System; SR, Saudi Riyal. * Low: less than high school; intermediate: high school or college; High: Bachelor’s degree or higher. ** Mixed: spastic and dyskinetic.

**Table 2:** Availability of the five major sources of support (*n* = 223).

Types of Available Support				
Source	Available		Not Available	
	*n*	%	*n*	%
Kinship	183	82.1	40	17.9
Spouse support	206	92.4	17	7.6
Informal support	175	78.5	48	21.5
Programs/organizations	108	48.4	115	51.6
Professional services	139	62.3	84	37.7

**Table 3:** Family Support Scale subscale mean scores (*n* = 223).

Source of Support	Mean	SD	Range	*p*-Value	ES
Kinship	2.3	1.8	0.0–5.0	<0.005 *	0.48
Spouse support	2.3	1.5	0.0–5.0		
Informal support	1.2	1.0	0.0–4.2		
Programs/organizations	0.5	0.8	0.0–3.8		
Professional services	0.9	1.1	0.0–5.0		
Informal social support	6.3	4.2	0.0–17.9	<0.005 *	0.64
Formal support	3.8	4.3	0.0–20.0		
Total support	24.3	17.7	0.0–78.0	-	

SD, standard deviation. ES, Kendall’s W effect size for Friedman test and effect size correlation coefficient (*r*) for Wilcoxon signed-rank test. Note: *p*-values were calculated using either Friedman test or Wilcoxon signed-rank test, depending on the number of categories. Informal social support represents the combined score of program organizations, informal support, spouse/partner support, and kinship subscales, whereas formal support represents the professional services subscale. * Significant at α = 0.05.

**Table 4:** Influence of child-related characteristics on levels of support (*n* = 223).

Characteristics	*n*	Informal Social Support				Formal Support				Total Support			
		M	SD	*p*-Value	ES	M	SD	*p*-Value	ES	M	SD	*p*-Value	ES
**Age** (years)													
<3	47	6.9	4.2	0.316	0.0	3.7	4.1	0.998	0.0	25.8	17.7	0.639	0.0
3 to <6	73	6.5	3.9			3.7	4.0			24.6	15.9		
6 to <12	84	5.8	4.4			3.9	4.7			23.3	18.7		
≥12	19	5.8	5.0			3.8	4.6			24.3	20.8		
**Gender**													
Boys	131	6.0	4.0	0.449	0.05	3.6	4.0	0.618	0.03	23.3	16.8	0.454	0.05
Girls	92	6.6	4.5			4.1	4.7			25.9	18.9		
**Type of CP**													
Ataxic	5	5.0	2.7	0.908	0.0	4.6	3.6	0.313	0.0	21.4	11.5	0.912	0.0
Dyskinetic	6	5.7	1.9			1.3	1.8			20.2	8.1		
Hypotonic	19	5.7	3.1			3.1	3.9			21.4	12.7		
Mixed	7	4.7	2.3			1.6	2.4			17.0	7.8		
Spastic	178	6.4	4.5			4.1	4.5			25.0	18.7		
Unknown	8	7.4	5.0			2.5	2.8			27.3	20.1		
**Distribution of CP** (n = 178)													
Unilateral	27	8.5	4.9	0.009 *	0.20	5.5	3.9	0.012 *	0.19	34.5	19.8	0.005 *	0.21
Bilateral	151	6.0	4.3			3.8	4.6			23.4	18.0		
**GMFCS group**													
I–III (Ambulatory)	123	7.0	4.5	0.010 *	0.17	4.5	4.7	0.018 *	0.16	27.5	18.8	0.007 *	0.18
IV–V (Non-ambulatory)	100	5.4	3.8			3.0	3.6			20.5	15.5		

M, mean; SD, standard deviation; ES, epsilon squared (*ε*^2^) effect size for Kruskal–Wallis *H* test and effect size correlation coefficient (*r*) for Mann–Whitney *U* test; CP, cerebral palsy; GMFCS, Gross Motor Function Classification System. Note: *p*-values were obtained using Kruskal–Wallis *H* test or Mann–Whitney *U* test, depending on the number of categories. Informal social support represents the combined score of program organizations, informal support, spouse/partner support, and kinship subscales, whereas formal support represents the professional services subscale. * Significant at α = 0.05.

**Table 5:** Influence of parents-related characteristics on levels of helpfulness of support (*n* = 223).

Characteristics	*n*	Informal Social Support				Formal Support				Total Support			
		M	SD	*p*-Value	ES	M	SD	*p*-Value	ES	M	SD	*p*-Value	ES
**Mother’s age** (years)													
21–30	72	7.4	4.3	0.023 *	0.03	3.9	4.6	0.749	0.0	27.4	18.5	0.325	0.01
31–40	99	6.0	4.2			3.5	4.0			23.3	17.7		
41–50	46	5.3	4.1			4.0	4.7			22.1	16.9		
>51	6	4.9	4.2			4.7	3.2			21.8	13.9		
**Mother’s education** **													
Low	42	5.3	4.6	0.075	0.02	3.3	3.8	0.713	0.0	21.0	19.0	0.149	0.01
Intermediate	97	6.1	4.0			4.0	4.6			24.0	17.4		
High	84	6.9	4.2			3.8	4.3			26.4	17.4		
**Mother’s employment**													
Unemployed	183	6.1	4.3	0.239	0.0	3.7	4.3	0.389	0.0	23.5	17.7	0.156	0.01
Part-time	5	9.4	5.3			6.0	5.8			39.2	24.3		
Full-time	35	6.5	3.9			4.2	4.5			26.7	16.5		
**Father’s age** (years)													
21–30	19	6.9	4.8	0.035 *	0.03	3.8	4.5	0.184	0.01	26.2	20.9	0.114	0.01
31–40	108	6.8	4.0			3.9	4.2			25.8	16.9		
41–50	69	5.2	4.0			3.0	3.7			20.4	16.2		
>51	27	6.3	5.0			5.5	5.8			27.2	21.4		
**Father’s education** **													
Low	36	5.6	5.0	0.245	0.0	4.4	5.5	0.960	0.0	23.0	22.1	0.412	0.0
Intermediate	111	6.4	3.8			3.6	3.9			24.4	15.8		
High	76	6.4	4.4			3.7	4.3			24.9	18.3		
**Father’s employment**													
Unemployed	44	5.9	4.5	0.621	0.0	4.8	5.2	0.504	0.0	24.4	19.3	0.996	0.0
Part-time	9	6.4	3.7			3.8	3.9			24.1	15.4		
Full-time	170	6.4	4.2			3.5	4.1			24.3	17.5		

M, mean; SD, standard deviation ES, epsilon squared (*ε*^2^) effect size. Note: *p*-values were obtained using Kruskal–Wallis *H* test. Informal social support represents the combined score of program organizations, informal support, spouse/partner support, and kinship subscales, whereas formal support represents the professional services subscale. * Significant at α = 0.05; ** Low: less than high school; intermediate: high school or college; high: Bachelor’s degree or higher.

**Table 6:** Influence of geographical and household characteristics on levels of support (*n* = 223).

Characteristics	*n*	Informal Social Support				Formal Support				Total Support			
		M	SD	*p*-Value	ES	M	SD	*p*-Value	ES	M	SD	*p*-Value	ES
**Region**													
Southern	51	7.4	5.8	<0.005 *	0.13	4.1	4.8	<0.005 *	0.10	28.1	24.3	<0.005 *	0.13
Central	49	8.4	3.8			5.6	4.9			33.4	17.1		
Eastern	28	4.1	1.4			1.3	1.5			15.6	5.7		
Western	88	5.4	3.3			3.7	3.9			21.2	13.4		
Northern	7	2.5	1.2			0.0	0.0			8.6	3.8		
**Living with** (n = 222) ^a^													
Mother	11	6.6	5.0	0.840	0.01	7.1	5.4	0.019 *	0.16	28.6	21.1	0.364	0.06
Both parents	211	6.2	4.2			3.6	4.2			24.1	17.6		
**Number of Children in the Same House**													
≤2	108	5.6	3.9	0.069	0.02	3.5	4.4	0.302	0.0	21.7	16.9	0.065	0.02
3–4	89	6.9	4.1			4.1	4.0			26.7	16.5		
≥5	26	6.9	5.5			3.8	5.1			27.2	23.2		
**Other Children with Special Needs in the Same House**													
No	190	6.5	4.4	0.142	0.10	4.1	4.4	0.019 *	0.16	25.5	18.2	0.028 *	0.15
Yes	33	5.1	3.3			2.3	3.6			17.8	13.0		
**Income** (SR)													
<5000	42	5.3	4.1	0.243	0.01	3.5	3.9	0.281	0.0	20.3	17.1	0.148	0.01
5000–10,000	97	6.3	4.0			3.4	4.1			23.9	16.7		
10,001–15,000	48	6.1	3.9			3.4	3.7			23.6	14.8		
>15,000	36	7.7	5.1			5.6	5.8			31.2	22.7		

M, mean; SD, standard deviation; ES, epsilon squared (*ε*^2^) effect size for Kruskal–Wallis *H* test and effect size correlation coefficient (*r*) for Mann–Whitney *U* test; SR, Saudi Riyal; Note: *p*-values were obtained using either Kruskal–Wallis *H* test or Mann–Whitney *U* test, depending on the number of categories. Informal social support represents the combined score of program organizations, informal support, spouse/partner support, and kinship subscales, whereas formal support represents the professional services subscale. * Significant at α = 0.05; ^a^ “Father” and “Others” categories were excluded due to an insufficient number of participants to perform the analysis.

## Data Availability

The data presented in this study are available on request from the corresponding author due to ethical and patient privacy considerations.

## Supplementary Materials

The following supporting information can be downloaded at: https://www.mdpi.com/article/10.3390/healthcare14101282/s1, Table S1. Results of pairwise comparisons for source of support scores (n = 223); Table S2. Results of pairwise comparisons of support levels based on geographical region (n = 223).

## Abbreviations

The following abbreviations are used in this manuscript:

CP	cerebral palsy
FSS	Family Support Scale
GMFCS	Gross Motor Function Classification System
