# Supplementary material for: Family-Centered Rehabilitation Care for Children with Cerebral Palsy in Saudi Arabia: Perceived Helpfulness of Formal and Informal Family Support
Source: Healthcare (Basel). 2026 May 8;14(10):1282. doi: 10.3390/healthcare14101282 (PMC13206552; doi:10.3390/healthcare14101282)
Supplement: Supplementary file 1 [file healthcare-14-01282-s001.zip › healthcare-4228768-supplementary.pdf]

Supplementary Table S1. Results of pairwise comparisons for source of support scores (n = 223).

Pairwise Comparison	*p*-value	ES
**Kinship vs. Spouse Support**	0.084	0.13
**Kinship vs. Informal Support**	< 0.005*	0.71
**Kinship vs. Programs/Organizations**	< 0.005*	0.85
**Kinship vs. Professional Services**	< 0.005*	0.72
**Spouse Support vs. Informal Support**	< 0.005*	0.80
**Spouse Support vs. Programs/Organizations**	< 0.005*	0.85
**Spouse Support vs. Professional Services**	< 0.005*	0.79
**Informal Support vs. Programs/Organizations**	< 0.005*	0.72
**Informal Support vs. Professional Services**	< 0.005*	0.32
**Programs/Organizations vs. Professional Services**	< 0.005*	0.57

ES, effect size correlation coefficient (*r*). Note: p-values were calculated using Wilcoxon signed-rank test. *Significant at $\alpha = 0.005$

Supplementary Table S2. Results of pairwise comparisons of support levels based on geographical region (n = 223).

Pairwise Comparison	Informal Social Support		Formal Support		Total Support	
	p-value	ES	*p*-value	ES	*p*-value	ES
**Southern vs. Central**	0.450	0.08	0.086	0.17	0.262	0.11
**Southern vs. Eastern**	0.164	0.16	0.133	0.17	0.375	0.10
**Southern vs. Western**	0.160	0.12	0.832	0.02	0.461	0.06
**Southern vs. Northern**	0.078	0.23	0.017	0.31	0.314	0.13
**Central vs. Eastern**	< 0.005*	0.59	< 0.005*	0.50	< 0.005*	0.63
**Central vs. Western**	< 0.005*	0.40	0.018	0.20	< 0.005*	0.38
**Central vs. Northern**	< 0.005*	0.50	0.001*	0.46	< 0.005*	0.54
**Eastern vs. Western**	0.058	0.18	0.002*	0.29	0.020	0.22
**Eastern vs. Northern**	0.011	0.43	0.011	0.43	0.005*	0.47
**Western vs. Northern**	0.012	0.26	0.003*	0.31	0.004*	0.29

ES, effect size correlation coefficient (*r*). Note: *p*-values were obtained using Mann–Whitney *U* test. Informal Social Support represents the combined score of Program Organizations, Informal Support, Spouse/Partner Support, and Kinship subscales, whereas Formal Support represents the Professional Services subscale. *Significant at $\alpha = 0.005$
